# Interactive Effects of LED Spectrum and Nitrogen Levels on Physiological Changes and Yield of Strawberry (*Fragaria × ananassa* Duch.)

**DOI:** 10.3390/plants14010089

**Published:** 2024-12-31

**Authors:** Sirajo Salisu Jibia, Kanokwan Panjama, Chaiartid Inkham, Takashi Sato, Norikuni Ohtake, Soraya Ruamrungsri

**Affiliations:** 1 Department of Plant and Soil Sciences, Faculty of Agriculture, Chiang Mai University, Chiang Mai 50200, Thailand; ssjibia@gmail.com (S.S.J.); guru_431@hotmail.com (K.P.); 2 Ph.D. Horticulture Program, Department of Plant and Soil Sciences, Faculty of Agriculture Under the CMU Presidential Scholarship, Chiang Mai University, Chiang Mai, 50200, Thailand; 3 Department of Agricultural Technology, Federal College of Agricultural Produce Technology, Kano 700223, Nigeria; 4 Economic Flower and Horticultural Crops Research Cluster, Chiang Mai University, Chiang Mai 50200, Thailand; sunwins111@hotmail.com; 5 H. M. The King’s Initiative Centre for Flower and Fruit Propagation, Chiang Mai 50230, Thailand; 6 Multidisciplinary Research Institute, Chiang Mai University, Chiang Mai 50200, Thailand; 7 Faculty of Bioresource Sciences, Akita Prefectural University, Akita 010-0195, Japan; t_sato@akita-pu.ac.jp; 8 Graduate School of Science and Technology, Niigata University, Niigata 950-2181, Japan; ohtake.agr@niigata-u.ac.jp

**Keywords:** LED, spectra, light, nitrogen, plant factory, strawberry

## Abstract

Strawberries are valued globally for their nutritional, aesthetic, and economic benefits. Optimizing blue-to-red LED ratios and nitrogen levels is essential for sustainable indoor strawberry cultivation. This factorial study investigated the effects of blue and red LED combination ratios (L1; 1:3, L2; 1:4, and L3; 1:6) and nitrogen levels (N1; 100 and N2; 200 mg/L) on the physiology and performance of strawberries in a plant factory. The results revealed that the interaction of L3 coupled with N2 maximized the vegetative growth of strawberry plants, whereas L2 and N2 produced the greatest biomass, while L2 interacted with N1 to expedite flowering. Photosynthesis and transpiration were enhanced by L3, particularly with 100 mg/L of nitrogen. The highest fruit yield and total soluble solids were obtained at the interaction of L3 and N1. Leaf nutrient analysis showed the highest nitrogen concentration at L1, while potassium increased with higher red LED ratios. The 100 mg/L nitrogen treatment resulted in higher leaf potassium concentrations than the 200 mg/L. These findings emphasize that LED spectra and nitrogen levels interact to optimize the physiology, vegetative and reproductive growth, maximizing fruit yield and quality in indoor strawberry cultivation. The study also concludes that the application of blue and red LED in the ratio of 1:6 with 100 mg/L nitrogen can improve indoor ‘Praratchatan 80’ strawberry performance.

## 1. Introduction

Garden strawberry (*Fragaria* × *ananassa* Duch.) is an important horticultural crop globally, owing to its high market and nutritional value. In 2021, the global production of strawberries reached 9,175,385.43 tons from 389,665 ha of farms [1]. Consumers favor strawberries due to their vibrant color, sweetness, and health benefits. The fruits are rich in bioactive compounds, including polyphenols, organic acids, and vitamin C, which possess antioxidant properties that may reduce oxidative stress and prevent chronic diseases [2,3]. Studies have associated strawberry consumption with the prevention of inflammatory disorders, reduction of obesity-related conditions, and cancer protection [2,4]. The demand for year-round production has led to the creation of advanced cultivation systems, such as plant factories with controlled environments (PFCE) that precisely regulate light and nutrients. Despite advancements in LED technology and nutrient optimization, the interaction between these factors in strawberry plants grown under PFCE conditions remains unclear.

In Thailand, strawberries are highly valued and are primarily grown in the cool mountainous regions of Chiang Mai and Chiang Rai, areas suitable for floral differentiation owing to cool temperatures at some times of the year [5]. The ‘Praratchatan 80’ cultivar, developed to suit local conditions, is notable for its disease resistance, large, sweet fruit, and popularity among fresh fruit consumers. This cultivar was obtained from a four-year breeding program using the Japanese Royal Queen cultivar developed by the Thai Royal Project Foundation [6].

Year-round strawberry cultivation in Thailand requires controlled-environment horticultural systems like plant factories for off-season urban production. These systems ensure a consistent supply, minimize logistical challenges, and reduce post-harvest losses caused by long-distance transport. Plant factories produce high-quality pest- and disease-free strawberries that appeal to consumers and command premium prices. Controlled environments extend the growing season, increase yield and quality, and enable multiple annual harvests. Additionally, plant factories reduce the need for pesticides and herbicides, resulting in cleaner, safer produce that aligns with organic and sustainable practices. Urban plant factories optimize land use through vertical farming, addressing land scarcity and cutting transportation costs to markets and consumption centers. They also allow for the cultivation of special strawberry varieties that are unsuitable for conventional farming due to their susceptibility to disease or harsh weather conditions. These innovations position Thailand as a leader in sustainable, high-tech fruit production.

Light energy is essential for photosynthesis and is often supplied artificially during controlled crop production, unlike sunlight in conventional cultivation. LEDs offer advantages over traditional light sources, including small size, long lifespan, high photoelectric conversion efficiency, and adjustable light spectra for plant growth [7]. LED lighting enhances photosynthesis, vegetative development, seed germination, and chlorophyll content of plants in controlled environments [8]. High-irradiance LED supplemental lighting is effective for high-yield strawberry cultivation, promoting growth and increasing fruit weight, number, and marketable yield [9]. Red-to-blue LED ratios are said to affect plant growth, with 20–30% blue light optimizing photosynthesis, whereas high blue light proportions can inhibit growth and reduce biomass [10,11,12,13,14]. Therefore, uncovering the optimum combination ratio of these two LED spectra can be game-changing in controlled environment strawberry cultivation.

Nitrogen is the most critical plant nutrient and is essential throughout the plant lifecycle from germination to senescence [15]. It also positively affects vegetative and reproductive traits of strawberry plants [16,17,18]. Different strawberry cultivars respond differently to different nitrogen levels. For instance, ‘Sweet Sensation’ and ‘Rubygem’ strawberry cultivars were reported to respond differently to nitrogen doses [17]. Nitrogen application increases plant height, number of leaves, canopy spread, flower and fruit number, yield, and total soluble solid (TSS) content [17,18]. The timing and amount of nitrogen application significantly influence growth, flowering time, and flower production [16,19]. However, excessive nitrogen application can lead to more vegetative growth at the expense of strawberry flowering and fruiting [20]. Thus, nitrogen optimization is key to resource-conscious and sustainable strawberry production.

While previous studies have explored the individual effects of LED spectra and nitrogen levels on strawberry performance, their combined impact on the ‘Praratchatan 80’ cultivar, particularly in plant factory settings, remains unexplored. This study hypothesized that specific combinations of red and blue LED light spectra and nitrogen levels could synergistically enhance strawberry performance in controlled systems. To address this, the study investigated the interactive effects of LED spectra and nitrogen levels on the physiology and yield of ‘Praratchatan 80’ strawberries grown in a plant factory setting. The findings aim to provide evidence-based insights into optimizing light and nitrogen inputs for sustainable strawberry cultivation. Furthermore, the results will serve as a foundation for future research and contribute to the development of tailored LED systems designed specifically for strawberry production.

## 2. Results

### 2.1. Growth Indices of Strawberry Plants

#### Number of Leaves, Plant Height, Crown Growth and Biomass Accumulation

**Figure 1:**
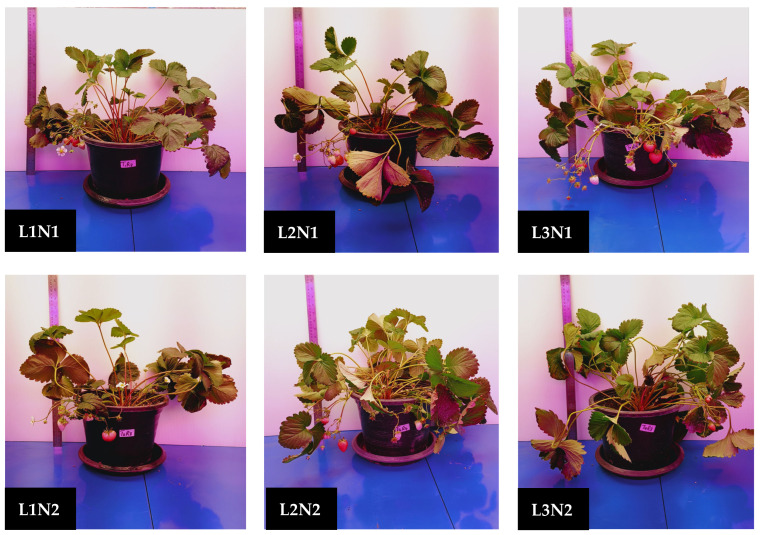
Strawberry plants during data collection, 8 weeks after treatment. L1, L2, and L3 represent the blue and red LED spectral combinations in the ratio of 1:3, 1:4, and 1:6, respectively. N1 and N2 refer to nitrogen treatments of 100 mg/L and 200 mg/L, respectively.

Data collected from strawberry plants (Figure 1) showed significant variations among the treatments. The LED spectra significantly (*p* < 0.05) influenced the growth parameters evaluated (Table 1). Among the treatments, L3 had the highest number of leaves ($\bar{x}$ = 29.85), plant height ($\bar{x}$ = 44.00 cm), and crowns per plant ($\bar{x}$ = 3.55). Conversely, L1 exhibited the lowest values for these parameters, particularly the number of leaves ($\bar{x}$ = 17.20) and plant height ($\bar{x}$ = 37.56 cm).

Nitrogen levels significantly affected the number of leaves and plant height (*p* < 0.05), whereas their effects on crowns per plant were not statistically significant (Table 1). Plants supplied with 200 mg/L nitrogen (N2) exhibited a greater number of leaves ($\bar{x}$ = 24.13) and greater height ($\bar{x}$ = 41.80 cm) than those treated with 100 mg/L nitrogen (N1).

Significant (*p* < 0.05) interactions between LED spectra and nitrogen levels were also observed. Notably, the combination of L3 and N2 yielded the highest number of leaves ($\bar{x}$ = 32.70), plant height ($\bar{x}$ = 45.14 cm), and number of crowns per plant ($\bar{x}$ = 3.70). In contrast, the L1 × N2 combination resulted in the lowest number of leaves ($\bar{x}$ = 15.80) and crowns per plant ($\bar{x}$ = 2.30).

The LED spectra significantly (*p* < 0.05) influenced both the total fresh and dry weights of strawberry plants (Table 1). Among the treatments, L2 yielded the highest fresh weight ($\bar{x}$ = 241.50 g) and dry weight ($\bar{x}$ = 48.40 g), followed by L3, which exhibited average weights of 206.93 g and 41.45 g for fresh and dry weight, respectively. The L1 treatment resulted in the lowest biomass production, with mean fresh and dry weights of 129.40 g and 25.88 g, respectively.

Nitrogen levels also significantly (*p* < 0.05) affected biomass accumulation (Table 1). Plants subjected to 200 mg/L nitrogen demonstrated greater fresh weight ($\bar{x}$ = 200.51 g) and dry weight ($\bar{x}$ = 40.11 g) than those exposed to 100 mg/L nitrogen, with mean fresh and dry weights of 184.76 g and 37.05 g, respectively.

Moreover, significant (*p* < 0.05) interactions between LED spectra and nitrogen levels were observed for both fresh and dry weights (Table 1). The combination of L2 and N2 produced the highest biomass, with fresh weight attaining a mean of 261.80 g and dry weight of 52.40 g. However, the L1 × N1 interaction resulted in the lowest biomass production, with fresh and dry weights of 127.56 g and 25.45 g, respectively.

### 2.2. Physiological Responses of Strawberry to LED Color Combinations and Nitrogen Levels

#### 2.2.1. Leaf Chlorophyll Index

The LED spectral combinations and nitrogen levels exhibited no significant (*p* < 0.05) differences in the SPAD readings. However, significant (*p* < 0.05) interactions between the LED spectra and nitrogen levels were observed for SPAD. The SPAD value was highest in L3 × N1 ($\bar{x}$ = 47.23) and on par with other combinations, indicating slight variations in chlorophyll content across treatments.

#### 2.2.2. Leaf Gas Exchange Attributes

Table 2 shows that LED spectral combinations significantly (*p* < 0.05) affected all measured photosynthesis parameters, including the net photosynthetic or carbon assimilation rate (Pn), transpiration rate (E), stomatal conductance (Gs), and intercellular CO₂ concentration (Ci). The highest Pn ($\bar{x}$ = 4.9579 µmol m⁻^2^ s⁻^1^) and E ($\bar{x}$ = 1.5007 mol m⁻^2^ s⁻^1^) occurred under the L3 treatment, while the highest Ci ($\bar{x}$ = 251.74 µmol mol⁻^1^) was recorded in the L2 treatment. The L1 treatment resulted in the lowest Ci ($\bar{x}$ = 232.89 µmol mol⁻^1^) and Pn ($\bar{x}$ = 2.6086 µmol m⁻^2^ s⁻^1^), indicating suboptimal performance compared to other spectral combinations.

Nitrogen levels significantly (*p* < 0.05) affected only Ci, with higher values in plants treated with 200 mg/L nitrogen ($\bar{x}$ = 246.46 µmol mol⁻^1^) compared to 100 mg/L ($\bar{x}$ = 238.49 µmol mol⁻^1^). No significant (*p* < 0.05) differences in Pn, E, or Gs were found among the nitrogen treatments (Table 2).

Significant (*p* < 0.05) interactions between LED spectra and nitrogen levels were observed for Pn, E, and Gs (Table 2). The L3 and N1 combinations yielded the highest Pn ($\bar{x}$ = 6.2971 µmol m⁻^2^ s⁻^1^) and E ($\bar{x}$ = 1.6914 mol m⁻^2^ s⁻^1^), while the L2 × N1 combination resulted in the lowest E ($\bar{x}$ = 0.9929 mol m⁻^2^ s⁻^1^).

### 2.3. Flower and Fruit Quality Characteristics

#### 2.3.1. Flowering Characteristics

LED spectral combinations significantly (*p* < 0.05) influenced all flowering attributes of strawberry plants, including days to first bloom, inflorescences per plant, flowers per plant, and florets per inflorescence (Table 3). The L3 treatment led to the shortest time to the first bloom ($\bar{x}$ = 38.90 days), while L2 resulted in the longest (41.45 days). L1 produced the most inflorescences ($\bar{x}$ = 2.20) and flowers per plant ($\bar{x}$ = 17.35), whereas L2 and L3 had fewer inflorescences ($\bar{x}$ = 1.60 and 1.55, respectively). The highest number of florets per inflorescence ($\bar{x}$ = 10.95) was found in L2, and the lowest ($\bar{x}$ = 8.23) occurred in L1.

Nitrogen levels significantly (*p* < 0.05) affected the days to first bloom and florets per inflorescence but not inflorescences and flowers per plant (Table 3). Plants receiving 100 mg/L nitrogen bloomed earlier ($\bar{x}$ = 37.40 days) than those given 200 mg/L ($\bar{x}$ = 42.70 days). N1 resulted in more florets per inflorescence ($\bar{x}$ = 10.58) than N2 ($\bar{x}$ = 8.59).

Significant (*p* < 0.05) interactions between LED spectra and nitrogen levels were observed for all flowering attributes (Table 3). The L2 and N1 combination led to the shortest time to bloom ($\bar{x}$ = 34.70 days) and the highest number of florets per inflorescence ($\bar{x}$ = 12.85). Conversely, the L2 × N2 combination delayed blooming the most ($\bar{x}$ = 48.20 days). The L1 × N2 combination resulted in the highest number of flowers per plant ($\bar{x}$ = 20.90), whereas the L3 × N2 combination produced the lowest ($\bar{x}$ = 10.60).

#### 2.3.2. Fruiting Characteristics

The LED spectral combinations significantly (*p* < 0.05) influenced all measured fruit attributes, including fruit number, weight, dimensions, shape index, total soluble solids (TSS), and firmness (Table 4).

Plants exposed to L2 produced the highest number of fruits per plant ($\bar{x}$ = 9.40), whereas plants exposed to L1 and L3 yielded significantly fewer fruits ($\bar{x}$ = 8.70). L2 and L3 equally improved the fruit yield by 71.28 g and 76.92 g, respectively, compared to L1. The largest fruits, as indicated by weight ($\bar{x}$ = 8.52 g) and dimensions (length: 30.22 mm, width: 27.07 mm) were observed under L3. Conversely, the smallest fruits ($\bar{x}$ = 5.57 g, 25.89 mm length, and 23.16 mm width) were associated with L1. The L3 treatment resulted in the highest TSS ($\bar{x}$ = 8.84 °Brix) and moderate firmness (4.55 N). In contrast, L1 fruits exhibited the highest firmness ($\bar{x}$ = 4.62 N) but the lowest TSS content ($\bar{x}$ = 6.93 °Brix). The shape index ($\bar{x}$ = 1.13) was consistent across L1 and L3, but slightly lower in L2 ($\bar{x}$ = 0.98).

Nitrogen levels significantly (*p* < 0.05) affected strawberry fruiting traits (Table 4). Plants that received 100 mg/L nitrogen produced more fruits per plant ($\bar{x}$ = 10.80) and higher fruit yield ($\bar{x}$ = 83.46 g) with greater weight ($\bar{x}$ = 7.55 g) and dimensions (length: 28.85 mm, width: 27.30 mm) than those treated with 200 mg/L nitrogen ($\bar{x}$ = 7.07 fruits per plant, 6.89 g, 25.13 mm, and 23.47 mm, respectively). Additionally, fruits from N1 plants demonstrated higher TSS ($\bar{x}$ = 8.14 °Brix) and firmness ($\bar{x}$ = 4.68 N) than those from N2 plants ($\bar{x}$ = 7.30 °Brix, 4.14 N).

**Figure 2:**
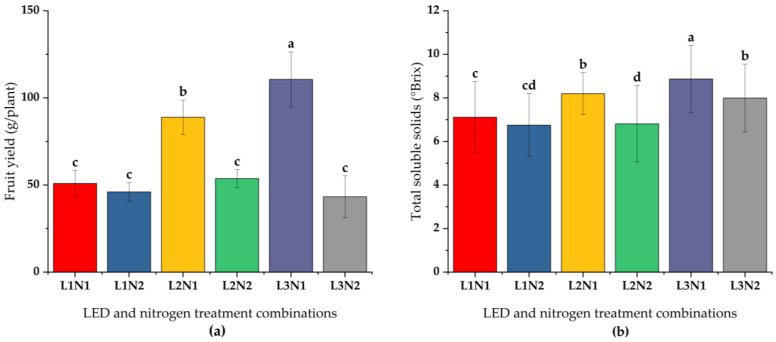
Interactive effects of blue (B; 400–500 nm) and red (R; 600–700 nm) LED combinations and nitrogen levels on fruiting of ‘Praratchatan 80’ strawberries under plant factory settings. Data was subjected to ANOVA (*p* < 0.05), and statistically significant means were separated using the Least Significant Difference (LSD, *p* < 0.05). (**a**) fruit yield of strawberry per plant, (**b**) total soluble solids content (Brix) in strawberry fruits. L1, L2 and L3; B:R LED ratio of 1:3, 1:4 and 1:6, respectively, N; 100 mg/L nitrogen and N2; 200 mg/L nitrogen, L1N1, …L3N2; interaction of respective blue and red LED ratios and nitrogen levels.

Furthermore, significant (*p* < 0.05) interactions between LED spectra and nitrogen levels were observed for all fruit traits (Table 4). The L3 × N1 combination resulted in the highest fruit yield ($\bar{x}$ = 11.80 fruits per plant), yield per plant ($\bar{x}$ = 110.62 g), and single fruit weight (Figure 2), whereas the L2 × N2 combination produced the fewest fruits ($\bar{x}$ = 7.20) with the smallest size ($\bar{x}$ = 21.58 mm length and 22.37 mm width). Moreover, the L3 × N1 treatment achieved the highest TSS ($\bar{x}$ = 9.11 °Brix, Figure 2), whereas the L2 × N2 treatment yielded the lowest ($\bar{x}$ = 6.58 °Brix). Firmness was highest under L2-N1 ($\bar{x}$ = 4.87 N) and lowest under L2 × N2 ($\bar{x}$ = 3.26 N).

### 2.4. Leaf Nutrient Contents

#### Total Nitrogen and Potassium Concentrations

The LED spectral combinations significantly (*p* < 0.05) influenced the total leaf nitrogen (%) and potassium (%) concentrations in the leaves and roots of ‘Praratchatan 80’ strawberry plants (Table 5).

The highest leaf nitrogen concentration ($\bar{x}$ = 2.79%) was recorded in L1, followed by L2 ($\bar{x}$ = 2.72%) and L3 ($\bar{x}$ = 2.53%). As for total potassium, L2 and L3 produced the highest potassium concentrations ($\bar{x}$ = 2.73% and 2.70%, respectively), whereas L1 had a significantly lower concentration ($\bar{x}$ = 2.53%).

Nitrogen levels significantly (*p* < 0.05) affected both nitrogen and potassium concentrations (Table 5). Strawberry plants treated with 100 mg/L nitrogen had higher total nitrogen ($\bar{x}$ = 2.76%) and potassium ($\bar{x}$ = 2.76%) concentrations than those treated with 200 mg/L nitrogen ($\bar{x}$ = 2.60% and 2.54%, respectively).

The interaction between the LED spectra and nitrogen levels significantly (*p* < 0.05) influenced the total nitrogen concentration (Table 5), while the interaction effect was not significant for the total potassium concentration. The L1 × N1 and L2 × N1 combinations resulted in the highest nitrogen concentrations ($\bar{x}$ = 2.83%), whereas the L3 × N2 combination yielded the lowest concentration ($\bar{x}$ = 2.45%). Although not statistically significant, the highest potassium concentration ($\bar{x}$ = 2.91%) was observed under L2 × N1, and the lowest ($\bar{x}$ = 2.40%) was recorded for L1 × N2.

## 3. Discussion

**Figure 3:**
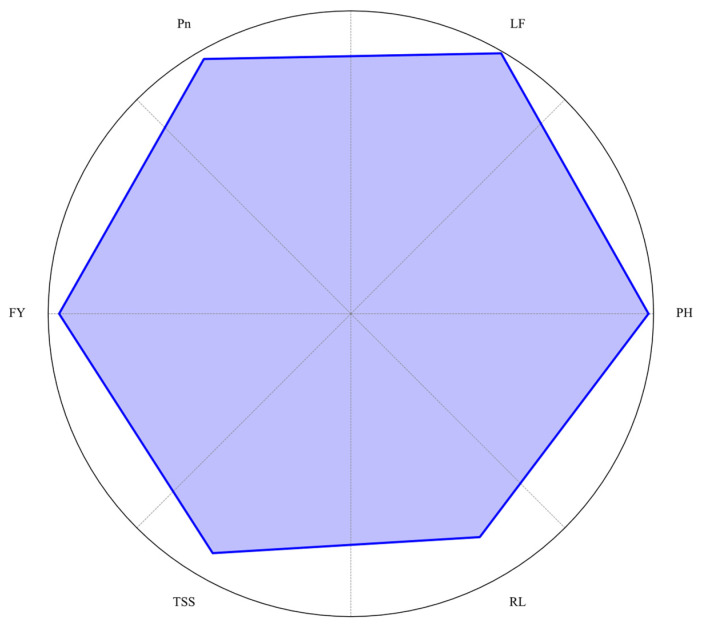
Radar chart summarizing the effects of the 1:6 blue-to-red LED light ratio combined with 100 mg/L nitrogen on plant performance parameters in strawberry (*Fragaria × ananassa* cv. ‘Praratchatan 80’). The parameters include plant height (PH), leaf number (LF), photosynthesis rate (Pn), fruit yield (FY), total soluble solids (TSS), and crown number (CN). Values were normalized to the maximum observed values for each parameter.

This investigation examined the interactive effects of three blue (400–500 nm) and red (600–700 nm) LED spectra (ratios of 1:3, 1:4, and 1:6) and two nitrogen levels (100 and 200 mg/L) on the growth, photosynthesis, flowering, fruiting, and nutrient dynamics of strawberry cv. ‘Praratchatan 80’ cultivated in a plant factory. The findings demonstrate that LED light quality and nitrogen levels independently and synergistically influence strawberry performance. Figure 3 represents the combined effects of 1:6 blue-to-red LED light ratio combined with 100 mg/L nitrogen, elucidating the potential to optimize these variables for high-quality, sustainable strawberry production. The study’s results provide valuable insights for developing precise management strategies in plant factories, potentially leading to more efficient and sustainable strawberry cultivation systems.

### 3.1. Growth Performance of Strawberry Plants

In our experiment, the combination of the 1:6 blue-to-red LED light ratio and 200 mg/L nitrogen resulted in the most substantial vegetative growth, including increased plant height, leaf number, and crown count of strawberry plants. These findings are consistent with previous studies demonstrating that red light promotes elongation and biomass accumulation, whereas blue light regulates leaf development and enhances chloroplast structure [11,12].

Notably, the 1:4 blue-to-red LED ratio treatment resulted in plants with the highest fresh and dry biomass, emphasizing the complementary role of red and blue light in facilitating photosynthesis and energy storage. Red light enhances photosystem II activity and ATP synthesis, whereas blue light improves chloroplast structure and stomatal conductance, enabling efficient uptake of water and nutrients [21,22]. These findings demonstrate that the red and blue light spectra are critical for photosynthesis, directly influencing plant growth and biomass production. It also indicates that increasing the ratio of red light might facilitate more vegetative growth at the expense of biomass accumulation.

However, nitrogen supplementation further augmented biomass accumulation, with 200 mg/L yielding superior results compared with 100 mg/L. However, this occurred at the expense of reproductive traits, consistent with previous studies suggesting that excessive nitrogen allocation favors vegetative growth over reproductive development [20]. This effect is likely due to increased cytokinin synthesis, which promotes cell division and expansion while delaying the transition to reproductive stages [23].

The synergy between the LED light spectra and nitrogen levels was evident. The 1:6 blue-to-red LED ratio treatment combined with 100 mg/L nitrogen promoted vegetative growth, whereas a 1:4 ratio combined with 200 mg/L nitrogen enhanced biomass production by 21.5% and 21.3% in fresh and dry weights, respectively. This synergy is attributed to physiological and molecular processes enhanced by both light quality and nitrogen. For instance, red light stimulates shoot growth and chlorophyll synthesis, whereas blue light improves stomatal conductance and root development, leading to enhanced water and nutrient uptake [24,25,26,27,28,29].

### 3.2. Photosynthesis

Exposure of strawberry plants to the 1:6 blue-to-red LED treatment resulted in the highest photosynthetic and transpiration rates despite lower biomass accumulation. This observation may be explained by the enhanced metabolite allocation to the fruit zone, leading to increased fruit weight under these conditions. While red light facilitates carbon fixation, blue light promotes stomatal conductance, chloroplast orientation, and photomorphogenesis, consequently improving gas exchange [11,30].

The combination of a 1:6 blue-to-red LED ratio and 100 mg/L nitrogen proved particularly advantageous, indicating its suitability for enhancing photosynthetic efficiency. In addition to promoting photosynthesis, red and blue light spectra improve nitrogen absorption and utilization, thereby further elevating photosynthetic rates [31,32,33,34]. Nitrogen augments the biochemical capacity for photosynthesis by increasing chlorophyll synthesis and Calvin cycle enzyme activity, thus complementing the effects of LED spectra [35,36,37,38].

Intricate physiological, molecular, and biochemical pathways orchestrate the synergistic effects of red and blue light and nitrogen levels. Genes associated with photosynthesis, nitrogen metabolism, and secondary metabolite biosynthesis are regulated by red and blue light, while nitrogen ensures the availability of adequate biochemical resources for these processes. This complex interplay highlights the potential of integrating optimized LED spectra with appropriate nitrogen levels to maximize strawberry growth and photosynthetic performance in controlled cultivation environments.

### 3.3. Flowering and Fruiting Characteristics

Our investigation revealed that the blue-to-red LED ratio of 1:6 expedited flowering while diminishing the number of inflorescences and florets. This LED configuration, when coupled with 100 mg/L nitrogen, yielded the heaviest fruits with the highest total soluble solids, emphasizing the significance of red light in augmenting sugar synthesis and nutrient distribution in fruits [22]. These results corroborate the findings of Liu, Lian [39], who noted enhanced fruit size and quality in strawberry plants exposed to red light. Reduced nitrogen concentrations promote fruiting by directing resources toward reproductive structures instead of vegetative growth.

Conversely, Kadowaki, Yano [40] demonstrated that blue light, in isolation, hastened flowering. An increased proportion of blue light, exemplified by a 1:3 red-to-blue light ratio, induces early flowering in diverse plant species, including Hippeastrum spp. and blueberries [41,42]. The combined effect of blue and red light amplifies photoperiodic signaling and carbohydrate accessibility, which are essential for flower bud differentiation and development [43]. Particular light spectra can also stimulate the expression of flowering genes such as FT, thereby accelerating flowering and enhancing fruit set [44,45]. Optimal light conditions also influence nutrient absorption necessary for floromorphogenesis; for example, red and blue light treatments facilitate the uptake of crucial nutrients, including nitrogen, potassium, magnesium, and iron [46,47,48].

The application of 100 mg/L nitrogen enhanced flowering characteristics by equilibrating vegetative and reproductive requirements. Elevated nitrogen levels (200 mg/L) delayed flowering due to increased vegetative growth, consistent with the findings of Iatrou and Papadopoulos [23]. The impact of nitrogen on flowering time exhibits a U-shaped response: both insufficient and excessive levels delay flowering, whereas an optimal concentration promotes it [49,50,51,52,53].

Nitrogen plays a crucial role in fruit development and affects fruit size, quality, and yield. Appropriate nitrogen levels boost photosynthesis, sugar formation, and carbohydrate metabolism, contributing to superior fruit sweetness, firmness, and nutritional content [18,54,55]. However, excessive nitrogen diminishes fruit firmness and increases susceptibility to disease [54,56]. The combination of red light and optimal nitrogen levels further enhances photosynthesis, secondary metabolite biosynthesis, and nitrogen assimilation [57,58,59,60,61].

To conclude, a 1:6 blue-to-red LED ratio, in conjunction with 100 mg/L nitrogen, improves flowering time and fruit yield in ‘Praratchatan 80’ strawberries. Red light enhances sugar synthesis and nutrient allocation, whereas blue light expedites flowering through photoperiodic signaling and carbohydrate availability. Collectively, these factors achieve a balance between vegetative and reproductive growth, culminating in enhanced fruit quality and yield.

### 3.4. Nutrient Concentrations

The highest leaf nitrogen concentration was observed under the 1:3 blue-to-red LED ratio, whereas the combination of 100 mg/L nitrogen and a 1:6 ratio resulted in the lowest concentration. This suggests that red light promotes nitrogen utilization by enhancing biomass accumulation, thereby reducing leaf nitrogen concentration. Blue light stimulates nitrate reductase activity and improves nitrate-to-ammonium conversion for protein synthesis [62].

Potassium concentration increased with a higher red-light ratio, reflecting its role in photosynthesis and enzyme activation. Red light facilitates stomatal conductance and carbohydrate metabolism, indirectly increasing potassium demand [63]. Lower nitrogen levels (100 mg/L) resulted in higher leaf potassium concentrations than higher nitrogen levels (200 mg/L), potentially because of an antagonistic relationship between nitrogen and potassium uptake.

The synergy between low nitrogen levels and potassium suggests a prioritization mechanism wherein potassium supports osmotic balance and photosynthetic efficiency under nitrogen-limited conditions. Combined blue and red LED light spectra enhance nutrient absorption, including nitrogen and potassium, thereby improving plant health and yield [32].

Nitrogen metabolism is particularly influenced by blue light, which enhances the activity and expression of nitrogen-related enzymes [27,64,65]. While red light alone reduces total nitrogen content, the combination of red and blue light optimizes nitrogen assimilation and enzyme activity, promoting protein synthesis [64,65].

Notably, higher nitrogen application (200 mg/L) reduced both nitrogen and potassium concentrations in the leaves. This contrasts with earlier findings that higher nitrogen levels generally increase the nitrogen content in plants. For instance, combining high nitrogen rates (10 mM N) with blue light significantly increases shoot nitrogen concentration and nitrogen nutrition index [27,66]. High nitrogen levels may alter allocation patterns within the plant, distributing nitrogen to other tissues rather than concentrating it in the leaves.

Optimal nitrogen levels and light spectra maximize nutrient concentrations and plant growth. A 1:4 blue-to-red LED ratio with 100 mg/L nitrogen enhanced the nitrogen and potassium concentrations in strawberry leaves. Adjusting the light quality and nitrogen levels concurrently optimizes strawberry nutrient uptake, particularly for nitrogen and potassium.

## 4. Materials and Methods

### 4.1. Experimental Design and Setup

#### 4.1.1. Treatments

**Figure 4:**
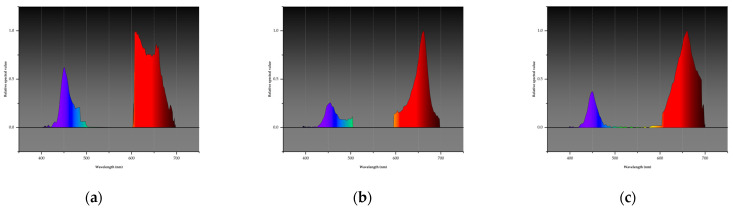
LED spectrum graphs displaying wavelengths corresponding to blue (B; 400–500 nm) and red (R; 600–700 nm) LED combinations used in the experiment. (**a**): L1 combination of blue and red light at a ratio of 1:3 (25% blue, 75% red); (**b**): L2, a combination at a ratio of 1:4 blue and red (20% blue and 80% red); and (**c**): L3 combination of blue and red colors at a ratio of 1:6 (14% blue, 86% red).

**Figure 5:**
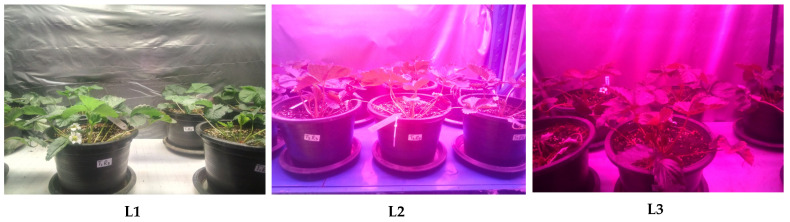
Strawberry plants under the different blue and red LED spectral combinations. (**L1**): a combination of blue and red light at a ratio of 1:3 (25% blue, 75% red); (**L2**): LED combination at a ratio of 1:4 blue and red (20% blue and 80% red); and (**L3**): a combination of blue and red colors at a ratio of 1:6 (14% blue, 86% red).

The experiment was conducted at the King’s Initiative Centre for Flower and Fruit Propagation, Chiang Mai, Thailand (18°42′50.148″ N, 98°55′15.06″ E) between 15 November 2023 and 15 February 2024. The trial followed a 3 × 2 factorial design in a completely randomized layout, with ten (10) replications (plants) per treatment. The LED spectra factor comprised three ratios of blue (B; 400–500 nm) and red (R; 600–700 nm) spectra: L1, B:R (1:3), L2, B:R (1:4), and L3, B:R (1:6) as represented in Figure 4 and Figure 5. Insights from previous research informed the selection of LED light ratios. For instance, Naznin, Lefsrud [67] reported that blue-to-red (B:R) ratios of 1:10 and 1:19 significantly enhanced fresh and dry mass accumulation in strawberry plants. Similarly, Guiamba, Zhang [68] demonstrated that a 30%:70% B:R ratio effectively promoted plant growth, while a study conducted in Japan evaluated B:R ratios of 1:2, 1:3, and 1:4. The findings indicated that the 1:3 and 1:4 ratios produced higher yields compared to the 1:2 ratio, with no significant differences between the 1:3 and 1:4 treatments [69]. Additionally, research on light spectra under stress conditions highlighted the efficacy of the 1:3 ratio in mitigating salinity and alkalinity stress, improving photosynthetic efficiency, and enhancing plant resilience [70].

Based on these and other findings, the decision to include the previously explored 1:3 and 1:4 ratios was aimed at re-examining their effects on strawberries in interactions with nitrogen treatments. Furthermore, the inclusion of a 1:6 ratio complements existing studies on red-dominant combinations, such as 1:10 and 1:19, by investigating the effects of incremental reductions in red light. Collectively, these selected ratios bridge the gap between substantially studied and relatively unexplored spectral combinations, addressing both extreme blue- and red-dominant conditions and contributing to the refinement of the current research on strawberry growth and productivity.

The nitrogen (N) factor consisted of two levels of 100 and 200 mg/L N (Table 6), initially applied in 50 mL and gradually increased to 250 mL nutrient solution per plant per day, with all other essential nutrients supplied equally. The nutrient solution was maintained at a pH of 6.00 and an EC of 1.5 dS/m. The experiment, focusing on flowering and fruiting, subjected the plants to nine hours of LED treatments daily throughout their growth period to induce a short-day condition.

#### 4.1.2. Plant Growth Conditions

Strawberry cv. ‘Praratchatan 80’ daughters, plants of the same age and size (at the three-leaf stage), were sourced from the Strawberry Multiplication Unit, Mae Hia Agricultural Training and Research Centre, Chiang Mai University. They were immediately moved to a cold chamber for vernalization at 5 °C for 14 days, as recommended by Thammasophon, Pusadee [6]. The plants were later transplanted into 7-inch plastic pots containing peat moss, perlite, and rice husks at a ratio of 2:1:0.5 and moved into the plant factory. Strawberry plants were cultivated under the following conditions: temperature, 25 ± 2 °C; relative humidity (RH) = 70–80%; carbon dioxide (CO_2_) concentration = 700–1000 ppm, and photosynthetic photon flux density (PPFD), ≈275 µmol m−^2^ s−^1^. In addition, all the emerging stolons were promptly removed.

### 4.2. Growth, Yield, and Gas Exchange Determination

Growth parameters were measured biweekly based on the number of leaves, plant height (cm), and number of crowns. Biomass accumulation was measured in terms of total plant fresh and dry weights (g) at harvest. Fresh weight was recorded after harvesting the plants, washing them with tap water, and rinsing them with deionized water, blotting them with paper towels, then allowing them to air dry for 20 min before being weighed using a precision balance. For dry weight, the already cleaned and dried plants were oven-dried for 72 h at 60–70 degrees Celsius and later weighed using a precision scale.

Flowering data were collected based on the date of first bloom, the number of inflorescences per plant, the number of florets per inflorescence, and the number of florets per plant.

Fruit characteristics and yield metrics, such as fruit count per plant, fresh fruit mass, fruit yield per plant, and total soluble solids (TSS), were recorded over the entire harvest period. TSS was measured with a PAL-1^®^ handheld refractometer (Atago Co., Ltd., Tokyo, Japan). Five fruits per plant were randomly selected and used for the Brix determination.

The net photosynthetic rate (Pn), stomatal conductance (Gs), and leaf transpiration rate (E) were measured using the LCPro-SD^®^ portable photosynthesis system (ADC BioScientific Ltd., Hoddesdon, Hertfordshire, United Kingdom). The data were recorded in the early mornings (10–11 am) from fresh but matured leaves (five leaves per plant). In contrast, leaf SPAD values were recorded using a Minolta ^®^ SPAD-502 chlorophyll meter (Konica Minolta, Inc., Tokyo, Japan) from the five topmost and fully expanded leaves per plant, and an average was recorded.

### 4.3. Nitrogen and Potassium Determination

Four sampled harvested strawberry plants were prepared using the procedure described in Section 4.2 above, then separated into leaves, shoots, roots, and reproductive parts. The leaves were oven-dried for 72 h at 60–70 degrees Celsius. The leaves were later finely ground using an electrical grinder and sifted through a fine mesh. The strawberry leaf powder was used for nitrogen and potassium determination. The total nitrogen (%) and potassium (%) concentrations in strawberry leaf tissues were determined at the Soil Science Laboratory, Department of Plant and Soil Sciences, Faculty of Agriculture, Chiang Mai University, Thailand. Nitrogen concentration was determined using Duma’s combustion procedures, as modernized by the Association of Official Analytical Chemists (AOAC) [71]. Total potassium was analyzed by atomic absorption spectrophotometry (AAS) as described by Mizukoshi, Nishiwaki [72].

### 4.4. Statistical Analyses

The collected data were analyzed using ANOVA, utilizing SciPy 1.0 [73] and supplementary libraries within the Python programming environment. Fisher’s Least Significant Difference (LSD) test was applied to identify significant differences among the treatment means at a 95% confidence level.

## 5. Conclusions

The present study investigated the interactive effects of LED spectra and nitrogen levels on physiological changes and yield of ‘Praratchatan 80’ strawberries under plant factory conditions. Findings from the study demonstrated that optimizing blue-to-red LED ratios and nitrogen levels can significantly enhance strawberry growth, photosynthesis, fruiting, and leaf nutrient concentrations. The findings indicated that a 1:6 blue-to-red LED ratio, in conjunction with 100 mg/L nitrogen, improves the balance between vegetative and reproductive growth, thereby maximizing fruit yield and quality. For controlled environment agriculture, these insights provide empirical information for refining LED light and nitrogen conditions to achieve sustainable, high-quality strawberry production. Further research is warranted to elucidate the molecular mechanisms underlying light-nutrient interactions and to investigate their applicability across diverse strawberry cultivars and environmental conditions.

## Figures and Tables

**Table 1:** LED spectra and nitrogen effects on ‘Praratchatan 80’ strawberry growth (90 DAP).

Factors	Number of Leaves	Height (cm)	Crowns per Plant	Fresh Weight (g)	Dry Weight (g)
LED spectra					
L1	17.20 ± 7.41 ^c^	37.56 ± 4.47 ^c^	2.50 ± 1.15 ^b^	129.40 ± 41.67 ^c^	25.88 ± 1.70 ^c^
L2	22.35 ± 5.20 ^b^	41.65 ± 3.45 ^b^	2.70 ± 0.73 ^b^	241.50 ± 55.61 ^a^	48.40 ± 4.79 ^a^
L3	29.85 ± 6.61 ^a^	44.00 ± 2.78 ^a^	3.55 ± 1.28 ^a^	206.93 ± 34.76 ^b^	41.45 ± 1.36 ^b^
Sig. (*p* < 0.05)	*	*	*	*	*
LSD	0.52	1.01	0.300	3.01	1.75
N Levels					
100 mg/L (N1)	22.13 ± 7.36 ^b^	40.33 ± 4.56 ^b^	2.87 ± 0.89	184.76 ± 51.90 ^b^	37.05 ± 8.72 ^b^
200 mg/L (N2)	24.13 ± 9.05 ^a^	41.80 ± 4.33 ^a^	2.97 ± 1.38	200.51 ± 75.94 ^a^	40.11 ± 11.25 ^a^
Sig. (*p* < 0.05)	*	*	ns	*	*
LSD	0.43	0.82	0.25	2.45	1.43
LED × N					
L1 × N1	18.60 ± 8.60 ^e^	35.64 ± 4.06 ^d^	2.70 ± 1.06 ^bc^	127.56 ± 35.44 ^d^	25.45 ± 2.37 ^d^
L1 × N2	15.80 ± 6.14 ^f^	39.47 ± 4.24 ^c^	2.30 ± 1.25 ^c^	131.24 ± 51.40 ^d^	26.28 ± 0.71 ^d^
L2 × N1	20.80 ± 4.26 ^d^	42.51 ± 2.66 ^b^	2.50 ± 0.71 ^bc^	221.36 ± 16.84 ^b^	44.39 ± 1.42 ^b^
L2 × N2	23.90 ± 5.80 ^c^	40.78 ± 4.06 ^c^	2.90 ± 0.74 ^b^	261.80 ± 75.20 ^a^	52.40 ± 3.07 ^a^
L3 × N1	27.00 ± 6.34 ^b^	42.85 ± 2.63 ^b^	3.40 ± 0.70 ^a^	205.36 ± 39.94 ^c^	41.26 ± 1.40 ^c^
L3 × N2	32.70 ± 5.83 ^a^	45.14 ± 2.55 ^a^	3.70 ± 1.70 ^a^	208.50 ± 33.42 ^c^	41.64 ± 1.47 ^c^
Sig. (*p* < 0.05)	*	*	*	*	*
CV (%)	3.56	8.38	16.23	1.69	4.92
LSD	0.74	1.42	0.42	4.25	2.48

*, significant (*p* < 0.05); ns, not significant; a–f, means followed by different superscript letters within a column are statistically different based on the LSD test (*p* < 0.05). DAP: days after planting.

**Table 2:** Photosynthetic traits of strawberry under blue-red LED spectra and nitrogen (90 DAP).

Factors	SPAD	Pn (µmol m⁻^2^ s⁻^1^)	E (mol m⁻^2^ s⁻^1^)	Gs (mol m⁻^2^ s⁻^1^)	Ci (µmol mol⁻^1^)
LED spectra					
L1	46.20 ± 3.13	2.61 ± 1.11 ^b^	1.40 ± 0.27 ^a^	0.15 ± 0.05 ^a^	232.89 ± 3.84 ^c^
L2	46.00 ± 3.30	3.80 ± 1.62 ^ab^	1.16 ± 0.22 ^b^	0.11 ± 0.04 ^b^	251.74 ± 4.16 ^a^
L3	46.72 ± 3.41	4.96 ± 2.11 ^a^	1.51 ± 0.29 ^a^	0.12 ± 0.04 ^b^	242.80 ± 4.00 ^b^
Sig. (*p* < 0.05)	ns	*	*	*	*
LSD	1.53	1.2358	0.2003	0.0298	3.07
N Levels					
100 mg/L	46.34 ± 3.06	4.15 ± 1.77	1.35 ± 0.26	0.12 ± 0.04	238.49 ± 3.94 ^b^
200 mg/L	46.27 ± 4.35	3.42 ± 1.46	1.36 ± 0.26	0.14 ± 0.04	246.46 ± 4.14 ^a^
Sig. (*p* < 0.05)	ns	ns	ns	ns	*
LSD	1.25	1.0090	0.1635	0.0244	2.51
LED × N Levels					
L1 × N1	46.90 ± 3.52 ^ab^	2.40 ± 1.02 ^b^	1.34 ± 0.25 ^b^	0.15 ± 0.05	230.24 ± 3.80
L1 × N2	45.52 ± 2.70 ^ab^	2.82 ± 1.21 ^b^	1.46 ± 0.27 ^ab^	0.15 ± 0.05	235.53 ± 3.87
L2 × N1	44.93 ± 1.70 ^b^	3.75 ± 1.61 ^b^	1.00 ± 0.19 ^c^	0.08 ± 0.03	247.80 ± 4.09
L2 × N2	47.07 ± 3.60 ^ab^	3.82 ± 1.63 ^b^	1.32 ± 0.25 ^b^	0.13 ± 0.04	255.67 ± 4.34
L3 × N1	47.23 ± 3.42 ^a^	6.30 ± 2.68 ^a^	1.69 ± 0.31 ^a^	0.13 ± 0.04	237.43 ± 3.95
L3 × N2	46.21 ± 1.32 ^ab^	3.62 ± 1.54 ^b^	1.31 ± 0.24 ^b^	0.12 ± 0.04	248.17 ± 4.17
Sig. (*p* < 0.05)	*	*	*	ns	ns
CV (%)	5.22	42.5900	19.3200	30.3400	1.65
LSD	2.17	1.7477	0.2833	0.0422	4.34

*, significant (*p* < 0.05); ns, not significant; a–c, means followed by different superscript letters within a column are statistically different based on the LSD test (*p* < 0.05). DAP: days after planting; Pn, net photosynthesis rate; E, transpiration rate; Gs, stomatal conductance; Ci, intercellular CO_2_.

**Table 3:** LED spectra and nitrogen effects on ‘Praratchatan 80’ strawberry flowering (90 DAP).

Factors	Days to First Bloom	Inflorescence per Plant	Flowers per Plant	Florets per Inflorescence
LED spectra				
L1	39.80 ± 1.88 ^b^	2.20 ± 0.62 ^a^	17.35 ± 3.77 ^a^	8.23 ± 0.62 ^c^
L2	41.45 ± 7.00 ^a^	1.60 ± 0.50 ^b^	16.80 ± 0.77 ^a^	10.95 ± 2.06 ^a^
L3	38.90 ± 2.90 ^c^	1.55 ± 0.51 ^b^	13.55 ± 13.55 ^b^	9.55 ± 1.40 ^b^
Sig. (*p* < 0.05)	*	*	*	*
LSD	0.78	0.31	0.56	2.19
N Levels				
100 mg/L	37.40 ± 3.08 ^b^	1.67 ± 0.48	15.80 ± 1.67	10.58 ± 2.10 ^a^
200 mg/L	42.70 ±4.24 ^a^	1.90 ± 0.71	16.00 ± 4.39	8.59 ± 0.71 ^b^
Sig. (*p* < 0.05)	*	ns	ns	*
LSD	0.64	0.26	0.46	0.36
LED × N				
L1 × N1	41.10 ± 1.45 ^b^	1.80 ± 0.42 ^b^	13.80 ± 0.79 ^c^	8.20 ± 0.63 ^d^
L1 × N2	38.50 ± 1.27 ^c^	2.60 ± 0.52 ^a^	20.90 ± 1.20 ^a^	8.31 ± 0.64 ^d^
L2 × N1	34.70 ± 0.95 ^e^	1.50 ± 0.53 ^b^	17.10 ± 0.74 ^b^	12.85 ± 0.58 ^a^
L2 × N2	48.20 ± 0.79 ^a^	1.70 ± 0.48 ^b^	16.50 ± 0.71 ^b^	9.05 ± 0.76 ^c^
L3 × N1	36.40 ± 1.78 ^d^	1.70 ± 0.48 ^b^	16.50 ± 0.97 ^b^	10.70 ± 0.95 ^b^
L3 × N2	41.10 ± 0.84 ^b^	1.40 ± 0.53 ^b^	10.60 ± 0.84 ^d^	8.40 ± 0.52 ^d^
Sig. (*p* < 0.05)	*	*	*	*
CV (%)	3.07	27.62	5.60	7.24
LSD	1.10	0.44	0.80	0.62

*, significant (*p* < 0.05); ns, not significant; a–e, means followed by different superscript letters within a column are statistically different based on the LSD test (*p* < 0.05). DAP: days after planting.

**Table 4:** Effect of varying LED spectral combinations and nitrogen levels on fruiting of strawberry.

Factors	Fruits per Plant	Weight (g)	Yield/Plant (g)	Length (mm)	Width (mm)	TSS (°Brix)	Firmness (N)
LED							
L1	8.70 ± 0.48 ^b^	5.57 ± 0.67 ^c^	48.46 ± 6.63 ^b^	25.89 ± 2.45 ^b^	23.16 ± 1.95 ^b^	6.93 ± 0.48 ^c^	4.62 ± 0.22 ^a^
L2	9.40 ± 2.36 ^a^	7.57 ± 0.73 ^b^	71.28 ± 20.01 ^a^	24.86 ± 4.22 ^b^	25.93 ± 5.23 ^a^	7.34 ± 0.88 ^b^	4.07 ± 0.83 ^c^
L3	8.70 ± 3.30 ^b^	8.52 ± 0.93 ^a^	76.93 ± 38.00 ^a^	30.22 ± 1.50 ^a^	27.07 ± 0.74 ^a^	8.84 ± 0.69 ^a^	4.55 ± 0.24 ^b^
Sig. (*p* < 0.05)	*	*	*	*	*	*	*
LSD	0.43	0.58	9.26	1.17	1.36	0.31	0.06
N Levels							
100 mg/L	10.80 ± 1.37 ^a^	7.55 ± 1.67 ^a^	83.46 ± 27.73 ^a^	28.85 ± 1.38 ^a^	27.30 ± 2.28 ^a^	8.14 ± 0.88 ^a^	4.68 ± 0.22 ^a^
200 mg/L	7.07 ± 1.28 ^b^	6.89 ± 1.18 ^b^	47.65 ± 8.80 ^b^	25.13 ± 4.38 ^b^	23.47 ± 370 ^b^	7.30 ± 1.10 ^b^	4.14 ± 0.67 ^b^
Sig. (*p* < 0.05)	*	*	*	*	*	*	*
LSD	0.35	0.48	7.56	0.95	1.11	0.65	0.05
LED × N							
L1 × N1	9.00 ± 0.00 ^b^	5.65 ± 0.83 ^c^	50.87 ± 7.50 ^c^	28.08 ± 0.39 ^b^	24.94 ± 0.61 ^c^	7.11 ± 0.28 ^c^	4.42 ± 0.10 ^c^
L1 × N2	8.40 ± 0.55 ^b^	5.50 ± 0.54 ^c^	46.04 ± 5.33 ^c^	23.69 ± 1.35 ^c^	21.37 ± 0.75 ^d^	6.75 ±c 0.58 ^cd^	4.83 ± 0.04 ^ab^
L2 × N1	11.60 ± 0.55 ^a^	7.65 ± 0.61 ^b^	88.89 ± 9.92 ^b^	28.15 ± 0.87 ^a^	29.73 ± 1.63 ^a^	8.20 ± 0.36 ^b^	4.87 ± 0.09 ^a^
L2 × N2	7.20 ± 0.45 ^c^	7.48 ± 0.90 ^b^	53.67 ± 5.18 ^c^	21.58 ± 3.58 ^d^	22.37 ± 4.80 ^d^	6.58 ± 0.26 ^d^	3.26 ± 0.11 ^d^
L3 × N1	11.80 ± 0.45 ^a^	9.35 ± 0.36 ^a^	110.62 ± 15.85 ^a^	30.31 ± 1.30 ^a^	27.23 ± 0.95 ^b^	9.11 ± 0.27 ^a^	4.76 ± 0.12 ^b^
L3 × N2	5.60 ± 0.55 ^d^	7.69 ± 0.32 ^b^	43.24 ± 11.95 ^c^	30.14 ± 1.76 ^a^	26.92 ± 0.45 ^b^	8.56 ± 0.87 ^b^	4.34 ± 0.12 ^c^
Sig. (*p* < 0.05)	*	*	*	*	*	*	*
CV (%)	5.21	8.75	9.30	6.82	8.47	6.35	2.23
LSD	0.61	0.82	13.10	1.65	1.93	0.44	0.09

*, significant (*p* < 0.05); ns, not significant; a–d, means followed by different superscript letters within a column are statistically different based on the LSD test (*p* < 0.05).

**Table 5:** Leaf nitrogen and potassium in ‘Praratchatan 80’ strawberry under LED spectra and nitrogen levels (90 DAP).

Factors	Total Nitrogen (%)	Total Potassium (%)
LED spectra		
L1	2.79 ± 0.08 ^a^	2.53 ± 0.22 ^b^
L2	2.72 ± 0.33 ^b^	2.73 ± 0.20 ^a^
L3	2.53 ± 0.09 ^c^	2.70 ± 0.14 ^a^
Sig. (*p* < 0.05)	*	*
LSD	0.05	0.15
N Levels		
100 mg/L	2.76 ± 0.12 ^a^	2.76 ± 0.20 ^a^
200 mg/L	2.60 ± 0.13 ^b^	2.54 ± 0.14 ^b^
Sig. (*p* < 0.05)	*	*
LSD	0.04	0.12
LED × N		
L1 × N1	2.83 ± 0.07 ^a^	2.67 ± 0.23 ^b^
L1 × N2	2.75 ± 0.07 ^b^	2.40 ± 0.12 ^c^
L2 × N1	2.83 ± 0.06 ^a^	2.91 ± 0.07 ^a^
L2 × N2	2.60 ± 0.03 ^c^	2.56 ± 0.08 ^bc^
L3 × N1	2.62 ± 0.03 ^c^	2.71 ± 0.21 ^ab^
L3 × N2	2.45 ± 0.02 ^d^	2.68 ± 0.05 ^b^
Sig. (*p* < 0.05)	*	ns
CV (%)	1.85	5.38
LSD	0.07	0.21

*, significant (*p* < 0.05); ns, not significant; a–d, means followed by different superscript letters within a column are statistically different based on the LSD test (*p* < 0.05). DAP: days after planting.

**Table 6:** Nitrogen treatments used in the experiment with a dilution ratio of 1:100.

Fertilizer Material	Concentration (g/L)	
	N1	N2
NH_4_NO_3_	21.10	49.80
KH_2_PO_4_	44.00	44.00
KNO_3_	19.10	19.10
CaCl_2_	20.00	20.00
MgSO_4_·7H_2_O	120.00	120.00
H_3_BO_3_	3.00	3.00
MnSO_4_·4H_2_O	2.00	2.00
ZnSO_4_·7H_2_O	0.22	0.22
CuSO_4_·5H_2_O	0.05	0.05
NaMoO_24_·2H_2_O	0.01	0.01
FeEDTA	1.50	1.50

N1; 100 mg/L, N2; 200 mg/L.

## Data Availability

All data upon which the conclusions of this study are based will be made available upon request. Requests should be directed to the corresponding author.
